# Distribution of polymorphic variants of CYP2A6 and their involvement in nicotine addiction

**DOI:** 10.17179/excli2016-847

**Published:** 2017-03-06

**Authors:** Luis A. López-Flores, Gloria Pérez-Rubio, Ramcés Falfán-Valencia

**Affiliations:** 1 Instituto Nacional de Enfermedades Respiratorias Ismael Cosio Villegas

**Keywords:** CYP2A6, ethnic differences, genetic polymorphism, tobacco consumption, nicotine metabolism, nicotine addiction

## Abstract

Tobacco consumption has become a major public health issue, which has motivated studies to identify and understand the biological processes involved in the smoking behavior for prevention and smoking cessation treatments. *CYP2A6* has been identified as the main gene that codifies the enzyme that metabolizes nicotine. Many alleles have been identified after the discovery of *CYP2A6*, suggesting a wide interethnic variability and a diverse smoking behavior of the allele carrying individuals. The main purpose of this review is to update and highlight the effects of the *CYP2A6* gene variability related to tobacco consumption reported from diverse human populations. The review further aims to consider *CYP2A6* in future studies as a possible genetic marker for the prevention and treatment of nicotine addiction. Therefore, we analyzed several population studies and their importance at addressing and characterizing a population using specific parameters. Our efforts may contribute to a personalized system for detecting, preventing and treating populations at a higher risk of smoking to avoid diseases related to tobacco consumption.

## Introduction

Tobacco consumption has become an epidemic affecting more than 1,000 million people worldwide and is considered the main cause of avoidable death causing approximately 6 million premature deaths each year (WHO, 2011[146]). Thus, tobacco consumption is a public health issue combined with economic losses; it has motivated studies for identifying and understanding the biological processes involved in the smoking behavior for prevention and smoking cessation treatments (Bierut et al, 2014[15]).

Among cigarette compounds, nicotine is responsible for causing dependence by stimulating the smoker and allowing the other compounds to access the body, causing chronic harmful effects and tobacco-related diseases.

Tobacco consumption is caused by environmental, psychosocial and genetic factors (Bierut, et al., 2014[15]). Previous studies have identified genes encoding proteins that influence nicotine addictive behavior due to their effect on the cerebral neurotransmission pathways (Al Koudsi and Tyndale, 2005[4]; Arinami et al., 2000[5]; Verde Rello and Santiago Dorrego, 2013[141]). Moreover, some gene products are involved in nicotine response as receptors and metabolizers (Hukkanen et al., 2005[55]; Verde Rello and Santiago Dorrego, 2013[141]). Pérez-Rubio et al. (2015[108]) have reviewed this subject in detail. In the metabolizers group, the *CYP2A6* gene plays an important role. Its protein product (by the same name) is the principal enzyme responsible for nicotine metabolism to cotinine and other sub-products in the human body. However, more than 45 alleles have been discovered suggesting wide interindividual and interethnic variety.

The main purpose of this review is to update and highlight the effects of the genetic polymorphisms of *CYP2A6* related to tobacco consumption reported in diverse human populations. Moreover, we aimed to consider these polymorphisms in future studies as possible genetic markers for the prevention and treatment of nicotine addiction.

## Biological Function of CYP2A6 in Smoking

The CYP2A6 enzyme belongs to the enzyme superfamily known as the cytochrome P450 system (CYP450), also classified in the drug metabolizing enzymes group. These enzymes are found in the endoplasmic reticulum of the cells of some tissues in the body, particularly in the liver. Moreover, they are phase I enzymes responsible for metabolizing more than 80 % of drugs such as xenobiotics and endogen products in the body (Evans and Relling, 2004[33]; Ingelman-Sundberg, 2004[56]).

CYP2A6 is found mainly in the liver and is approximately 5-10 % of the total CYP450 content (Shimada et al., 1996[120]; Yamano et al., 1990[148]), although it has also been found to be expressed in the nasal mucosa, trachea, bronchi, lungs, sinuses and several brain regions (Bhagwat et al., 2000[14]; Chiang et al., 2012[22]; Crawford et al., 1998[24]; Ding and Kaminsky, 2003[29]; Macé et al., 1998[78]).

CYP2A6 has demonstrated to be involved in the metabolism of some endogen and exogen substrates such as precarcinogenic and carcinogenic compounds, as well as some toxins and drugs including nicotine.

CYP2A6 has been particularly important in tobacco consumption because of its involvement in nicotine metabolism. Nicotine is the main compound in tobacco responsible for the development of cigarette addiction by stimulating nicotinic cholinergic receptors (nAChR) that release neurotransmitters in the brain and cause a pleasant sensation in the smoker. The nicotine availability in the body is mediated by biological factors, mainly those related to its metabolism. Smokers tend to consume the same amount of nicotine each day to acquire the desired effects by modulating their smoking behavior to adjust nicotine availability for the purpose of regulating nicotine levels in the body (Benowitz, 1992[10]).

It has been reported that CYP2A6 is the main enzyme involved in the nicotine oxidation to cotinine. CYP2A6 catalyzes approximately 80 % (55-92 %) of this reaction via C-oxidation in addition to other metabolic pathways for nicotine and its metabolites (Benowitz and Jacob III, 1994[11]; Messina et al., 1997[83]; Nakajima et al., 1996[94]). Some other enzymes of CYP450 contribute to a lesser degree to nicotine metabolism such as CYP2B6, CYP2A13, CYP2D6 and CYP2E1 (mentioned in the order of relevance).

These biological products interact directly with nicotine to affect physiological brain processes (e.g., nAChR) and are inactivated and removed from the body (e.g., CYP450 enzymes) making their genes ideal candidates for altering smoking behavior (Malaiyandi et al., 2005[82]).

## CYP2A6 Genetic Variants

The *CYP2A6* gene has a 6 kb extension length, and it is composed of 9 exons, which encode for a 494 amino-acid product (Fernandez-Salguero et al., 1995[34]). It is located in the chromosomal band 19q13.2, where other CYP450 gene subfamilies (*CYP2B*, *CYP2F*, *CYP2G*, *CYP2S*, and *CYP2T*) are also present. The CYP2A subfamily cluster includes the *CYP2A6*, *CYP2A7*, and *CYP2A13* genes and other pseudogenes (Hoffman et al., 2001[52]).

The *Human CYP-Allele Nomenclature Database* (HCAND) (http://www.cypalleles.ki.se) was created in 1999 for the identification and characterization of *CYP2A6* alleles (and other CYP450 genes). This database consists of a committee for unifying and assigning nomenclature for the already discovered alleles and alleles to be discovered in the future (Sim and Ingelman-Sundberg, 2010[121], 2013[122]). 

**Figure 1:**
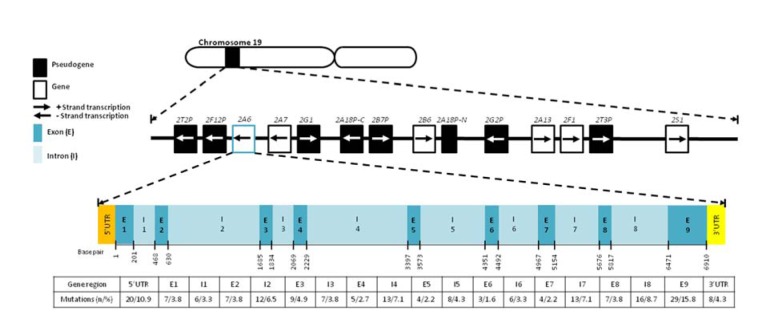
*CYP2A6* location and mutations in the gene. Mutations are reported by the *Human CYP-Allele Nomenclature Database *(http://www.cypalleles.ki.se) and include insertion, deletion, CNV and SNP. Ref Seq NG_008377.1

To date, 42 well-characterized alleles and some haplotypes that are uncharacterized (“CYP2A6 allele nomenclature,” 2014[25]) have been identified. These alleles are determined according to the origins of their mutation(s), such as gene conversion, gene deletion, gene duplication and/or single nucleotide polymorphism (SNP). The gene mutations are summarized in Figure 1(Fig. 1).

The wild-type allele that is considered as a reference is CYP2A6*1A (Yamano et al., 1990[148]). CYP2A6*1B consists of a 58 bp gene conversion with *CYP2A7* in the *CYP2A6 *3' UTR region (Ariyoshi et al., 2000[8]; Yamano et al., 1990[148]). *In vitro *and* in vivo a*ssays have shown that the *CYP2A6 *3' UTR region has more enzymatic activity and RNA stabilization than the reference allele (Ho et al., 2009[49]; Wang et al, 2006[143]; Yoshida et al., 2002[150]). However, several haplotypes contain this gene conversion and other noncoding genetic changes and have been designated CYP2A6*1B1-B17 (Ariyoshi et al., 2000[8]; Haberl et al., 2005[46]; Mwenifumbo et al., 2007[89], 2008[87], 2010[90]; Nakajima et al., 2006[91]; Pitarque et al., 2004[111]; Yamano et al., 1990[148]). Moreover, it has been determined that other wild-type alleles, named CYP2A6*1D-L, that have genetic changes in coding, noncoding and regulatory regions (Mwenifumbo et al., 2008[87], 2010[90]; Nakajima et al, 2004[95]; Pitarque et al., 2004[111]; von Richter et al., 2004[142]; Yamano et al., 1990[148]).

CYP2A6*2 consists of a missense mutation of 1799T>A, causing an amino acid change of Leu160His in the enzyme. Thus, the protein does not incorporate the heme group, inactivating the enzyme for *in vitro* and *in vivo* assays (Benowitz et al., 1995[12]; Hadidi et al., 1997[47]; Oscarson, et al., 1999[103]; Yamano et al., 1990[148]).

CYP2A6*3 is presumed to be a gene conversion of *CYP2A6* to *CYP2A7*, which results in an inactive enzyme; however, the methodology for their detection, function, and allelic frequency has been controversial (Fernandez-Salguero et al., 1995[34]; Oscarson et al., 1998[100]; Yamano et al., 1990[148]).

CYP2A6*4 consists of a homologous unequal crossover with *CYP2A7* on several positions (CYP2A6*4A-F), which leads to a whole gene deletion causing loss of enzymatic activity (Kitagawa et al., 1999[62]; Nakajima et al., 2000[93], 2001[92]; Nunoya et al., 1999[97]).

CYP2A6*5 contains a missense mutation, 6582G>T, creating a Gly479Val amino acid change and resulting in a lack of enzyme function (Oscarson et al., 1999[103]).

CYP2A6*6 contains a missense mutation, 6582G>T, which creates an Arg128Gln amino acid change causing lower enzymatic activity of the enzyme (Kitagawa et al., 2001[63]).

CYP2A6*7 contains a missense mutation, 6558G>A, which produces an Ile471Thr amino acid change that decreases the enzymatic activity to metabolize some substrates for *in vivo* and *in vitro* assays (Ariyoshi et al, 2001[7]; Uno et al., 2013[138]; Xu et al., 2002[147]).

CYP2A6*8 contains a missense mutation, 6600G>T, creating an amino acid change in Arg485Leu; however, the mutation's effect apparently does not change the enzymatic activity (Xu et al., 2002[147]).

CYP2A6*9 contains a point mutation, -48T>G, on the TATA box located in the gene promoter, which results in a decrease of more than 50 % of the enzymatic activity for *in vitro *and* in vivo* assays (Pitarque et al., 2001[110]; Yoshida et al., 2003[149]). There have been identified two subtypes of this allele: CYP2A6*9A, which contains an additional -1013A>G point mutation (von Richter et al., 2004[142]), and CYP2A6*9B, which contains the point mutations -1680A>G, -1301A>C, -1289G>A, 1620T>C, 1836G>T, 6354T>C and 6692C>G (Haberl et al., 2005[46]).

CYP2A6*10 contains two point mutations, similar to the CYP2A6*7 and CYP2A6*8 alleles. These point mutations decrease the enzymatic activity dramatically and make it completely inactive for some substrates (Xu et al., 2002[147]).

CYP2A6*11 contains a missense mutation, 3391T>C, which results in the amino acid change of Ser224Pro, decreasing the enzymatic activity (Daigo et al., 2002[26]).

CYP2A6*12A originated by the unequal crossover between *CYP2A6 *and* CYP2A7*, which resulted in a hybrid allele at the 5' UTR and exons 1- 2 belonging to *CYP2A7* and from the 3^rd^ to 9^th^ exon belonging to *CYP2A6*. This generates a 10 amino acid substitution compared to the reference allele (Oscarson et al., 2002[101]). Later, several SNPs were discovered in the same allele, which generates two subvariants (CYP2A6*12B-C) (Haberl et al., 2005[46]). These alleles are classified as null enzymatic activity (Bloom et al., 2011[17]).

CYP2A6*13 has two point mutations: the first at -48T>G in the TATA box of the promoter and the second at 13G>A changes the amino acid Gly5Arg. The enzymatic activity is decreased (Kiyotani et al., 2002[64]; Nakajima et al., 2006[91]).

CYP2A6*14 has the missense mutation 86G>A and changes the Ser29Asn amino acid chain; however, this does not affect the enzymatic activity (Kiyotani et al., 2002[64]; Nakajima et al., 2006[91]).

CYP2A6*15 is the product of two point mutations: the first at -48T>G in the TATA box of the promoter and the second at 2134 A>G, which results in an amino acid change of Lys194Glu (Kiyotani et al., 2002[64]). However, this enzyme does not show differences in substrate metabolism (Tiong et al., 2014[134]; 2010[135]).

CYP2A6*16 has a missense mutation at 2161C>A, which makes an amino acid change of Arg203Ser (Kiyotani et al., 2002[64]); however, it does not cause a defect in the enzymatic activity (Ho et al., 2008[50]; Nakajima et al., 2006[91]; Tiong et al., 2014[134], 2010[135]).

CYP2A6*17 shows several point mutations, 51G>A, 209C>T, 1779G>A, 4489C>T, 5065G>A, 5163G>A, 5717C>T and 5825A>G, which result in the amino acid change Val365Met and cause a decrease in the enzymatic activity of the allele (Fukami et al., 2004[41]).

CYP2A6*18 has three subvariants that share the missense mutation at 5886A>T and the amino-acid change Tyr392Phe. CYP2A6*18A only has that point mutation while CYP2A6*18B also has synonymous substitutions at 51G>A, 5684T>C and 5702T>C. CYP2A6*18C also has the point mutations at -1680A>G, -1579T>C, -1464A>T, -1301A>C, -1289G>A, -1013A>G, 1620T>C, 5668A>T and 6692C>G. The enzymatic activity of this allele tends to be specific according to the substrate (Fukami et al., 2005[38]; Haberl et al., 2005[46]).

CYP2A6*19 is produced by the point mutations at 5668A>T, 6354T>C, and 6558T>C and a gene conversion at the 3'UTR with *CYP2A7*, which correspond to the amino acid changes of Tyr392Phe and Ile471Thr, decreasing the enzymatic activity (Fukami et al., 2005[38]).

CYP2A6*20 has a frameshift mutation at nucleotides 2140 and 2141, which displaces the frameshift from the codon 196 to stop prematurely at 220 codons. In addition, it has three point mutations at 51G>A, 5684T>C and 6692C>G. These mutations produce a loss-of-function enzyme (Fukami et al., 2005[37]; Mwenifumbo et al., 2008[87]).

CYP2A6*21 is the result of two point mutations: 51G>A and 6573A>G, which produce an amino acid change Lys476Arg (Haberl et al., 2005[46]). However, the functional effect of the enzyme is still under discussion because it has been reported that *in vivo *assays show differences according to the study population (Al Koudsi et al., 2006[3]; Mwenifumbo et al., 2008[87]) and *in vitro* assays show normal enzymatic activity (Tiong et al., 2014[134], 2010[135]).

CYP2A6*22 is the result of three point mutations: 51G>A, 1794C>G and 1798C>A, which cause the amino acid changes Asp158Glu and Leu160Ile (Haberl et al., 2005[46]). These mutations reduce the enzyme affinity to the substrates; CYP2A6*22 has 39 % of the enzyme activity compared to the reference allele (Tiong et al., 2014[134], 2010[135]).

CYP2A6*23 contains a point mutation at 2161C>T that corresponds to the amino acid change Arg203Cys; this decreases the enzymatic activity to 19 % compared to the reference allele (Ho et al., 2009[49], 2008[50]).

CYP2A6*24 has two subvariants, among which CYP2A6*24A has the following point mutations: -1301A>C, -1289G>A, -1013A>G, 51G, 578A>G, 594G>C, 720G>A, 1137C>G, 1620T>C, 2483G>A, 3225A>G, 5668A, 6218A>G, 6282A>G, 6293T>C, 6354T>C, 6458A>T, , 6782C>G and 7160A>G, as well as a gene conversion in the 3'UTR of 58 bp. However, CYP2A6*24B has a 1381_1382CT>TC substitution and 1481_1486delCTCTCT deletion. These mutations cause the amino acid changes Val110Leu and Asn438Tyr, which encode a loss-of-function enzyme (Mwenifumbo et al., 2008[87]).

CYP2A6*25 is the result of several point mutations, some of them in 5'UTR: -1301A>C, -1289G>A and -745A>G, also 22C>T, 51G, 768A>T, 1620T>C, 1672T>C, 2296C>T, 2483G>A, 2605G>A, 2921G>A, 2994T>C, 4636A>C, 5668A, 6586T>C, 6692C>G and 7160A>G that result in the amino acid change of Phe118Leu (Mwenifumbo et al., 2008[87]), it has been shown that its enzymatic activity is decreased in some substrates (Ho et al., 2009[49]; Mwenifumbo et al., 2008[87]; Uno et al., 2013[138]).

CYP2A6*26 is produced by the following point mutations: -1301A>C, -1289G>A, -745A>G, 22C>T, 51G, 1165G>A, 1620T>C, 1672T>C, 1703G>T, 1710C>T, 1711T>G, 2296C>T, 2483G>A, 2994T>C, 4071delA, 4636A>C, 5668A, 6115C>T, 6586T>C, 6692C>G and 7160A>G which make amino acid changes in Phe118Leu, Arg128Leu and Ser131Ala (Mwenifumbo et al., 2008[87]). The *in vivo* and *in vitro* assays have proven that the final product is a loss-of-function enzyme (Ho et al., 2009[49]; Mwenifumbo et al., 2008[87]).

CYP2A6*27 has the following point mutations: -1301A>C, -1289G>A, -745A>G, 22C>T, 51G, 1620T>C, 1672T>C, 2162_2163GC>A, 2296C>T, 2483G>A, 2994T>C, 3872G>A, 4071delA, 4636A>C, 5668A, 5857T>A, 6586T>C, 6692C>G and 7160A>G, which makes the amino acid change Phe118Leu and also have a frameshift mutation, which displaces the frameshift to stop prematurely at 5^th^ exon (Mwenifumbo et al., 2008[87]), resulting in a loss-of-function enzyme (Ho et al., 2009[49]; Mwenifumbo et al., 2008[87]).

CYP2A6*28 has two subvariants that share the following mutations: -1269T>C, 51G>A, 656G>T, 1620T>C, 4681T>G, 5668A, 5738C>T, 5745A>G, 5750G>C, 6354T>C, 6361C>A and 7160A>G, as well as a gene conversion in the 3'UTR of 58 bp. However, CYP2A6*28A also carries the point mutations 6385G>T, 6389C>G, 6390T>C and 6782C>G, on the other hand CYP2A6*28B has additionally the following mutations: 1381_1382CT>TC, 6960_6961ins GAAAAG and 1481_1486delCTCTCT. These mutations cause the amino acid change of Asn418Asp and Glu419Asp, however, the enzymatic activity is the same as the reference allele (Mwenifumbo et al., 2008[87]).

According to the HCAND, CYP2A6*29,* 30*,* 32 *and* 33* alleles are now in evaluation phase (“CYP2A6 allele nomenclature,” 2014[25]).

CYP2A6*31 has two subvariants which share the following mutations: -1289G>A, -1013A>G, -492_-470delCCCCTTCCTGAGACCCTTAACCCinsAATCCATATGTGGA ATCTG, 16A>C, 51G, 1339C>G, 1620T>C, 2721G>A, 2994T>C, 3255A>G, 3315C>T, 5668A, 6692C>G and 7160A>G. However, CYP2A6*31A also has the point mutation of 7568C>T and CYP2A6*31B the mutations in -975T>C, 467C>T and 4074delA that cause the amino acid change Met6Leu (Mwenifumbo et al., 2008[87]), but the enzymatic activity has not been evaluated.

CYP2A6*34 originated by the unequal crossover between *CYP2A6 *and* CYP2A7,* which resulted in a hybrid allele at the 5'UTR and exons 1- 4 belonging to *CYP2A7 *and from the 5^th^ to 9^th^ exon belonging to *CYP2A6* (di Iulio et al., 2009[28]). The enzymatic activity has not yet been evaluated, however could be similar to CYP2A6*12 and it might be a loss-of-function allele.

CYP2A6*35 has two subvariants that share the following mutations: -1301A>C, -1289G>A, 1620T>C, 6458A>T, 6782C>G, 7160A>G and a gene conversion at the 3'UTR with *CYP2A7*. However, CYP2A6*35A also has the following point mutations: -1013A>G, 720G>A, 1137C>G, 2483G>A, 3225A>G, 6218A>G, 6282A>G, 6293T>C and 6354T>C; on the other hand, CYP2A6*35B has the following point mutations: -745A>G, 22C>T, 4084delA, 6835C>A and 6999T>C (Al Koudsi et al., 2010[2]). These mutations produce a protein that has the amino acid change Asn438Tyr which decrease the enzymatic activity according to *in vitro* and *in vivo* assays (Al Koudsi et al., 2010[2]).

CYP2A6*36 has the following point mutations: -1301A>C, -1289G>A, -745A>G, 22C>T, 1620T>C, 4084delA, 6458A>T, 6558T>C, gene conversion at 3'UTR, 6782C>G, 6835C>A, 6999T>C and 7160A>G that change the amino acid of Asn438Tyr and Ile471Thr (Al Koudsi et al., 2010[2]). To date there are no assays that prove their enzymatic activity.

CYP2A6*37 is produced by the following mutations: -1301A>C, -1289G>A, -745A>G, 22C>T, 1620T>C, 4084delA, 6354T>C, 6458A>T, 6558T>C, 6600G>T, 6782C>G, 6835C>A, 6936_6937insCACTT, 6961_6962insGAAAAG, 6989A>G, 6999T>C, 7160A>G and a gene conversion at the 3'UTR, which make the amino acid changes Asn438Tyr, Ile471Thr and Arg485Leu (Al Koudsi et al., 2010[2]). There are no assays that prove the enzymatic activity.

CYP2A6*38 is a result of the missense mutation 5023A>G, which produces the amino acid change of Tyr351His (Bloom et al., 2011[17]). An in silico assay classified the SNP as harmful, suggesting a decreased enzymatic activity (Bloom et al., 2011[17]).

CYP2A6*39 was described by Pilinguian et al. (2014[109]) as a missense mutation of 468G>A; however, the HCAND (“CYP2A6 allele nomenclature,” 2014[25]) adds the point mutations 171C>A, 1779G>A and 5717C>T, which cause the amino acid change Val68Met. The enzymatic activity of this allele is reported as decreased to half of the reference allele (Piliguian et al., 2014[109]).

CYP2A6*40 has the missense mutation 1767C>G (Piliguian et al., 2014[109]), but later the HCAND (“CYP2A6 allele nomenclature,” 2014[25]) added the point mutations 144G>A, 3492C>T and 5738C>T, which modifies the amino acid to Ile149Met. The enzymatic activity is reported to be half of the reference allele (Piliguian et al., 2014[109]).

CYP2A6*41 contains the missense mutation 3515G>A according to Pilinguian et al. (2014[109]), but the HCAND (“CYP2A6 allele nomenclature,” 2014[25]) added the point mutations 51G>A and 507C>T, which changed the amino acid to Arg265Gln. This allele was shown to have a minimal alteration in expression and a normal enzymatic activity (Piliguian et al., 2014[109]).

CYP2A6*42 is the result of a missense mutation 3524T>C according to Pilinguian et al. (2014[109]), but the HCAND (“CYP2A6 allele nomenclature,” 2014[25]) added the mutations 51G>A and 5684T>C, which made the amino acid change Ile268Thr that decreases the expression and enzymatic activity on *in vivo* and *in vitro* assays (Piliguian et al., 2014[109]).

CYP2A6*43 has the missense mutation 4406C>T, which makes the amino acid change to Thr303Ile and shows decreased expression and enzymatic activity in *in vivo* and *in vitro* assays (Piliguian et al., 2014[109]).

CYP2A6*44 carries the missense mutation 5661G>A according to Pilinguian et al. (2014[109]), but later the HCAND (“CYP2A6 allele nomenclature,” 2014[25]) added the mutations 51G>A, 5738C>T, 5745A>G and 5750G>C, which modify the amino acids to Glu390Lys, Asn418Asp and Glu419Asp. These mutations have been shown to reduce the enzymatic activity and the reference allele expression to one-third (Piliguian et al., 2014[109]).

CYP2A6*45 has a missense mutation at 6531T>C according to Pilinguian et al. (2014[109]), but later the HCAND (“CYP2A6 allele nomenclature,” 2014[25]) added the point mutations 51G>A and 4464G>A, which change the amino acid Leu462Pro. The mutations have been proven (as in CYP2A6*44) to reduce the enzymatic activity and the reference allele expression to one-third (Piliguian et al., 2014[109]).

There are two *CYP2A6* gene duplications: CYP2A6*1X2A originated by an unequal crossover from the 8^th^ to 9^th^ exon with CYP2A6*4D as a reciprocal product (Rao et al., 2000[112]). CYP2A6*1X2B also originated by an unequal crossover of *CYP2A7* from 5.2 to 5.6 kb downstream of the stop codon with CYP2A6*4B as the reciprocal product (Fukami et al., 2007[40]). Its enzyme activity has been shown to increase in *in vivo* assays (Rao et al., 2000[112]).

*CYP2A6* has been suggested as a highly polymorphic gene because it is located in a small chromosomal region that contains several genes and some unequal crossover events, point mutations and genetic conversions between *CYP2A6* and* CYP2A7* (Hoffman et al., 1995[51]). These facts, plus evolutionary forces such as genetic drift and natural selection, may have resulted in this genetic variability, which spread among human populations (Ingelman-Sundberg, 2004[56], 2005[58]). This genetic variability, similar to other CYP450 genes, could explain the metabolic response to exogenous compounds such as nicotine and other drugs that ranges from 20-40 % (Ingelman-Sundberg, 2001[57]).

*CYP2A6* genotypes can be classified according to their alleles and their enzymatic activity, which is referred to as the metabolism range of 3-hydroxycotinine/cotinine (Dempsey et al., 2004[27]), as mentioned below:

-Ultrarapid metabolizers. Individuals who have an enzymatic activity >100 % of normal; they contain more than two functional alleles (the CYP2A6*1X2 allele).

-Normal metabolizers. Individuals who have an enzymatic activity of 100 % (normal); they contain two functional alleles.

-Intermediate metabolizers. Individuals who have an enzymatic activity ≤75 % of normal may contain a functional and a defective allele or even two partially defective alleles.

-Slow metabolizers. Individuals who have an enzymatic activity ≤50 % of normal can contain a functional and a loss-of-function allele or even two loss-of-function alleles.

## Effects of CYP2A6 Genetic Variants on Tobacco Consumption

The genetic variability of CYP2A6 directly influences the range of nicotine metabolism in the body, which can indirectly affect the reinforcing and aversive nicotine properties in the brain and can change the individual risk of nicotine dependence. To prove the effect of *CYP2A6 *variants on tobacco consumption, several studies were conducted that included family, twin and non-related subject designs. These studies associate an allele with the amount of metabolized nicotine or another variable related to tobacco consumption.

Smokers who carry some *CYP2A6* alleles show a different smoking behavior compared to carrying the wild-type allele, suggesting that smokers regulate their smoking behavior to obtain the desired nicotine levels in their body (Malaiyandi et al., 2005[82]; Rao et al., 2000[112]; Schoedel et al., 2004[117]).

The importance of the null and decreased biological function alleles is explained in smokers (carrying these alleles) who exhibit less time smoking (Liu et al., 2011[74]; Malaiyandi et al., 2006[81]), fewer cigarettes smoked (Audrain-McGovern et al., 2007[9]; Fujieda et al., 2004[36]; Malaiyandi et al., 2005[82]; Minematsu et al., 2006[84]; O'Loughlin et al., 2004[99]; Pan et al., 2015[104]; Rao et al., 2000[112]; Schoedel et al., 2004[117]; Thorgeirsson et al., 2010[132]), fewer aspirations per cigarette (Strasser et al., 2011[125]), later smoking onset (Gu et al., 2000[43]; O'Loughlin et al., 2004[99]; Schoedel et al., 2004[117]) and less nicotine dependence (Wassenaar et al., 2011[144]). Likewise, it has been reported that these individuals respond better to replacement nicotine therapy (Lerman et al., 2010[72]; Malaiyandi et al., 2006[81]). Moreover, they tolerate withdrawal symptoms (Kubota et al., 2006[68]) better and have higher rates of quitting smoking spontaneously (Chenoweth et al., 2013[21]; Malaiyandi et al., 2006[81]; Minematsu et al., 2003[85]; Ray et al., 2009[113]). These alleles have also been associated with lung, bladder, nasopharyngeal, esophageal and oral cancer (Fujieda et al., 2004[36]; Hosono et al., 2015[53]; Islam et al., 2013[59]; Ito et al., 2015[60]; Kumondai et al., 2016[69]; Lourembam et al., 2015[77]; Miyamoto et al., 1999[86]; Song et al., 2009[123]; Tamaki et al., 2011[130]; Tan et al., 2001[131]; Tiwawech et al., 2006[136]; Topcu et al., 2002[137]; Wassenaar et al., 2015[145]).

On the other hand, some reports do not prove the association between null and decreased *CYP2A6* alleles related to tobacco consumption (London et al., 1999[75]; Loriot et al., 2001[76]; Sabol and Hamer, 1999[116]; Schulz et al., 2001[118]; Tiihonen et al., 2000[133]; Zhang et al., 2001[153]). This lack of association may occur because of several factors such as designing the population stratification (comparing populations where there are substructures between cases and controls) and population ethnicity, lack of detailed phenotypic evaluation, indeterminate comorbidities, different genotyping methods, examination of different allelic variants, inconsistency in smoking history and differences in symptoms of nicotine dependence among smokers (Lerman and Niaura, 2002[73]; O'Loughlin et al., 2004[99]).

Detecting the alleles of *CYP2A6 *can allow us to characterize different smoking behaviors and smoking-related diseases among individuals (Fujieda et al., 2004[36]), due to their role in nicotine metabolism and the metabolism of certain carcinogenic compounds. This could have an implication on public health by reducing the harmful effects related to smoking and developing a personalized smoking cessation according to their individual genotype (Liu et al., 2011[74]; Schoedel et al., 2004[117]). However, these alleles have a specific distribution in worldwide populations.

## Population Distribution of CYP2A6 Alleles

The *CYP2A6* allele distribution has an interethnic pattern. Knowing the individual differences in nicotine metabolism may allow us to answer the following questions: Why do some people become regular smokers after initial exposure, while others experience negative reactions and discontinue use? Why do some people smoke in greater quantities than others? Why do different individuals not respond the same way to drug therapies to quit smoking? Why do some individuals develop smoking related diseases faster than others? (O'Loughlin et al., 2004[99]; Schoedel et al., 2004[117]; Swan et al, 1997[128], 2005[127]).

**Figure 2:**
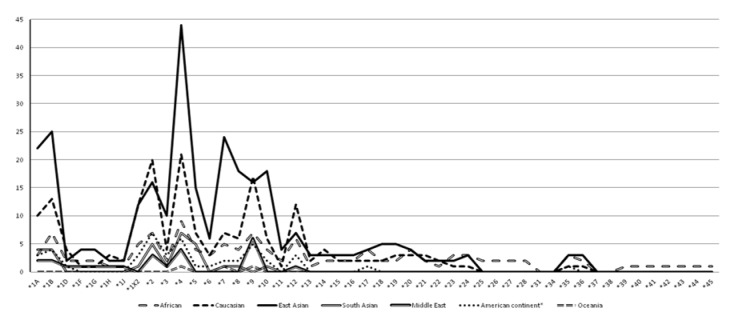
*CYP2A6* alleles reported in tobacco consumption related studies. Number of *CYP2A6* alleles that have reported frequencies in population and association studies related to tobacco/nicotine consumption and cancer related to tobacco consumption (in the population study). The populations are grouped according to possible ancestral and / or geographical origin: African (Canadian, American, Ghanaian, Ethiopian, Namibian, “African” and “Black” populations). Caucasian (German, Canadian, American, Spanish, Finish, French, Hungarian, English, Serbian, Swedish, “Caucasian” and “Whites”). East Asian (Chinese, Korean, Japanese, Malaysia, Thai, Taiwanese, Vietnamese, Han Chinese, Uighur, Bouyei, Tibetan, Shimane, Tottori, Fukuoka, Ehime and “Asian”) South Asian (Indian, Bangladeshi, Sri Lanka, Tamilian, Kannadika, Keralites and Andhrites). Middle East (Iranian, Turkish, Tatar, Turks, Turkomans and Zoroastrian Persians). American Continent (Hispanic, Brazilian, Chilean, Ecuadorian, Mexican, Canadian Native and Alaskan Yupik). Oceania (Neo Zealander Māori). *Except African American, American and Canadian.

Therefore, we present the allele frequencies in reported populations where tobacco consumption, cancer and nicotine metabolism were studied and in cohorts and general population studies, which involve *CYP2A6*. The number of reports of each allele according to a population group is summarized in Figure 2(Fig. 2). For more practical reasons, we only showed the frequency without distinction for alleles with subvariants, except for the wild-type allele CYP2A6*1 whose frequency was not completely calculated because some studies assign an unidentified genotype to the reference allele.

The wild-type allele subvariants are distributed in a particular way on worldwide populations. CYP2A6*1A is found in almost all populations studied such as Caucasian populations (Spanish, British, French, Swedish and Serbian), which range from 57-67 % (Djordjevic et al., 2010[30], 2013[31]; Gambier et al., 2005[42]; Huang et al., 2005[54]; Nakajima et al., 2006[91], 2004[95]; Oscarson, et al., 1999[102]; Soriano et al., 2011[124]), Africans, and Ethiopians, which reported an allele frequency of 34.8 % (Aklillu et al., 2014[1]). However, in African Americans, Ghanaians and Namibians the frequencies were between 66.5-80.5 % (Gyamfi et al., 2005[45]; Nakajima et al., 2004[95]; Takeshita et al., 2006[129]) while it was the opposite in Asians. Asians living in the UK report a frequency of 64.1 % while East and South Asian populations (Chinese, Japanese, Korean, Malaysian, Thai, Indian, Bangladeshi and Sri Lankan) report a frequency of 27-52 % (Ariyoshi et al., 2002[6]; Djordjevic et al., 2013[31]; Islam et al., 2013[59]; Ito et al., 2015[60]; Iwahashi et al., 2004[61]; Kwon et al., 2001[70]; Mahavorasirikul et al., 2009[79]; Nakajima et al., 2001[92], 2006[91]; Nurfadhlina et al., 2006[98]; Oscarson et al., 1999[102]; Peamkrasatam et al., 2006[107]; Takeshita et al., 2006[129]; Topcu et al., 2002[137]; Yoshida et al., 2002[150]). In the Middle East, the Turkish report a frequency of 23.9-69.7 % (Takeshita et al., 2006[129]; von Richter et al. (2004[142]). The American mestizo populations such as Brazilian and Ecuadorian show a frequency of 71.7 % (Rossini et al., 2006[115]) and 61.7 % (Soriano et al., 2011[124]), respectively. CYP2A6*1B frequency is higher in East and South Asian populations and is reported as 26.7-54.2 % (Ariyoshi et al., 2002[6]; Djordjevic et al., 2013[31]; Islam et al., 2013[59]; Ito et al., 2015[60]; Iwahashi et al., 2004[61]; Kwon et al., 2001[70]; Lourembam et al., 2015[77]; Mahavorasirikul et al., 2009[79]; Nakajima et al., 2001[92], 2006[91]; Nurfadhlina et al., 2006[98]; Oscarson et al., 1999[102]; Peamkrasatam et al., 2006[107]; Schoedel et al., 2004[117]; Takeshita et al., 2006[129]; Tiwawech et al., 2006[136]; Topcu et al., 2002[137]; Yoshida et al., 2002[150]; Yusof and Gan, 2009[152]). In Caucasians (Nonspecific, North American, Spanish, British, French, Swedish and Serbian) a frequency of 27.6-33.5 % (Bloom et al., 2011[17]; Djordjevic et al., 2013[31], 2010[30]; Gambier et al., 2005[42]; Haberl et al., 2005[46]; Huang et al., 2005[54]; Nakajima et al., 2006[91], 2004[95]; Oscarson et al., 1999[102]; Schoedel et al., 2004[117]; Soriano et al., 2011[124]) is reported, but it is lower in Turkish populations (25.9-26.7 % (Takeshita et al., 2006[129]; von Richter et al., 2004[142])). African populations such as African American, Ghanaian and Namibian show lower frequencies (11.2-19.8 % (Gyamfi et al., 2005[45]; Ho et al., 2009[49]; Mwenifumbo et al., 2008[87]; Nakajima et al., 2006[91], 2004[95]; Schoedel et al., 2004[117]; Takeshita et al., 2006[129])), except for Ethiopian populations (31.3 % (Aklillu et al., 2014[1])). American mestizo populations (Brazilian and Ecuadorian) have a frequency of 26.4-31.2 % (Rossini et al., 2006[115]; Soriano et al., 2011[124]; Vasconcelos et al., 2005[139]) while the Native Canadian and Alaskan Yupik show a frequency of 55-65.3 % (Rossini et al., 2006[115]; Soriano et al., 2011[124]; Vasconcelos et al., 2005[139]). CYP2A6*1D showed a higher frequency in the African nonspecific population (>50 % (Nakajima et al., 2006[91])) than in the Ethiopian population (29.4 %) (Aklillu et al., 2014[1]). In the Caucasian population (Nonspecific, North American and Swedish) the frequency is 26.7-40 % (Bloom et al., 2011[17]; Nakajima et al., 2006[91]; von Richter et al., 2004[142]) and in Turkish populations it is 32.3 % (von Richter et al., 2004[142]). Asian populations (Japanese and Korean) show a frequency of 10-20 % (Nakajima et al., 2006[91]). CYP2A6*1F has been reported only in nonspecific Caucasian (1.8 % (Nakajima et al., 2004[95])) and Turkish (2.2 % (Takeshita et al., 2006[129])) populations, but was not found in African and Japanese populations (Nakajima et al., 2004[95]; Takeshita et al., 2006[129]). CYP2A6*1G has a higher frequency in African populations (North American and Namibian) (12.3-13.3 % (Nakajima et al., 2004[95]; Takeshita et al., 2006[129])) than in Caucasian populations (1.2 % (Nakajima et al., 2004[95])), but it was not reported in Turkish and Japanese populations (Takeshita et al., 2006[129]). CYP2A6*1H has been found higher in Caucasian populations such as nonspecific Caucasian (>11 % (Nakajima et al., 2006[91])), North American (7.9 % (Bloom et al., 2011[17])) and Swedish (3.1 % (von Richter et al., 2004[142])), followed by nonspecific African (9.8 % (Nakajima et al., 2006[91])), Turkish (5.2 % (von Richter et al., 2004[142])), Japanese (4.5 % (Nakajima et al., 2006[91])) and Korean (1.6 % (Nakajima et al., 2006[91])). CYP2A6*1J has been reported as non-existent in Caucasian, African, Japanese and Korean populations (Nakajima et al., 2006[91]).

CYP2A6*2 has a higher frequency in Caucasian populations such as nonspecific Caucasian (1.1-5.3 % (Audrain-McGovern et al., 2007[9]; Benowitz et al., 2006[13]; Haberl et al., 2005[46]; Malaiyandi et al., 2006[80][81]; Nakajima et al., 2006[91], 2004[95]; Paschke et al., 2001[106]; Rao et al., 2000[112]; Schoedel et al., 2004[117]; Xu et al., 2002[147])), English populations such as British (2.5 % (Huang et al., 2005[54])) and Canadian (3.4 % (O'Loughlin et al., 2004[99])); and in Central Europe populations such as German, French and Spanish it has been reported from 2.3-3 % (Bourian et al., 2000[19]; Loriot et al., 2001[76]; Oscarson, et al., 1999[103])). In North of Europe populations such as Finish and Swedish, a frequency of 1.1-3 % (Oscarson et al., 1998[100], 1999[103]) was reported. In African populations (Afro American and Ethiopian), the frequency is less than 1 % (Malaiyandi et al., 2005[82]; Nakajima et al., 2006[95]; Schoedel et al., 2004[117]) and is not reported in Ghanaian populations (Gyamfi et al., 2005[45]). The frequency is different in American populations: Amerindian populations, Canadian natives and Alaskan Yupik have a frequency less than 1 % (Binnington et al., 2012[16]; Nowak et al., 1998[96]; Schoedel et al., 2004[117]). However, the mestizo populations such as Brazilian (1.6-1.7 %) (Rossini et al., 2006[115]; Vasconcelos et al., 2005[139]) and Chilean (2-3.7 %) (Cáceres et al., 2012[20]; Roco et al., 2012[114]) show a frequency higher than Hispanics (Benowitz et al., 2006[13]). In East and Southeast Asian populations such as Chinese, Korean, Japanese, Malaysian and Thai, CYP2A6*2 is non-existent (Huang et al., 2005[54]; Kitagawa et al., 1999[62]; Malaiyandi et al., 2005[82]; Nakajima et al., 2006[91]; Nurfadhlina et al., 2006[98]; Oscarson et al., 1999[103]; Schoedel et al., 2004[117]). A minimum range of 1 % (Nurfadhlina et al., 2006[98]) was reported in Indians. The Iranian population had a frequency higher than 2.2 % (Emamghoreishi et al., 2008[32]; Heravi et al., 2010[48]), unlike the Turkish population where CYP2A6*2 was not found (Cok et al., 2001[23]).

The methodologies for the detection, function and allelic frequency of CYP2A6*3 have been controversial (Fernandez-Salguero et al., 1995[34]; Oscarson et al., 1998[100]; Yamano et al., 1990[148]); however, we present the reported frequency. The highest frequency (13.8 % (Nowak et al., 1998[96])) was reported in Canadian natives, followed by Turkish (12 % (Cok et al., 2001[23])), German (1.4 %) (Bourian et al., 2000[19]) and Indian (1.1 %) (Nurfadhlina et al., 2006[98]) populations. However, in African (Nakajima et al., 2006[91]; Paschke et al., 2001[106]). Caucasian (Nakajima et al., 2006[91]; Paschke et al., 2001[106]), Spanish (Oscarson et al., 1999[102]), Chilean (Cáceres et al., 2012[20]), Iranian (Heravi et al., 2010[48]), Korean (Kwon et al., 2001[70]; Nakajima et al., 2006[91]; Yoshida et al., 2002[150]), Chinese (Nurfadhlina et al., 2006[98]; Oscarson et al., 1999[103]), Malaysian (Nurfadhlina et al., 2006[98]) and Japanese (Nakajima et al., 2001[92], 2006[91]; Yoshida et al., 2002[150]) (except a report of 0.2 % (Minematsu et al., 2003[85])) populations, this allele was not found. 

CYP2A6*4 may be the most widely studied allele in all populations. East Asian populations such as Japanese (11.2-25.6 % (Ariyoshi et al., 2002[6]; Fujieda et al., 2004[36]; Fukami et al., 2006[39]; Ito et al., 2015[60]; Iwahashi et al., 2004[61]; Minematsu et al., 2003[85], 2006[84]; Nakajima et al., 2001[92], 2006[91]; Schoedel et al., 2004[117]; Takeshita et al., 2006[129]; Tamaki et al., 2011[130]; Xu et al., 2002[147]; Yoshida et al., 2002[150])), Chinese (4.9-14 % (Gu et al, 2005[44]; Liu et al., 2011[74]; Nurfadhlina et al., 2006[98]; Oscarson et al., 1999[102]; Schoedel et al., 2004[117]; Song et al., 2009[123]; Tan et al., 2001[131]; Xu et al., 2002[147]; Yuan et al., 2016[151])), Korean (9.4-11 %) (Djordjevic et al., 2013[31]; Fukami et al., 2006[39]; Kwon et al., 2001[70]; Nakajima et al., 2006[91]; Yoshida et al., 2002[150]), Thai (4-14.2 % (Mahavorasirikul et al., 2009[79]; Peamkrasatam et al., 2006[107]; Tiwawech et al., 2006[136])), Malaysian (7-16.7 % (Nurfadhlina et al., 2006[98]; Yusof and Gan, 2009[152])) and Vietnamese (11.8 % (Veiga et al., 2009[140])) showed the highest frequency among populations. However, some Asian populations that reside in a foreign country such as those living in the UK report a frequency of less than 1 % (Benowitz et al., 2006[13]; Huang et al., 2005[54]). However, ethnic populations in Asian countries show no differences in the frequencies such as in Japan with the Shimane (18.2 %), Tottori (16.9 %), Fukoka (20.6 %) and Ehime (25.9 % (Takeshita et al., 2006[129]) and in China with the Han (7.9 %), Uighur (15 %), Bouyei (0 %) and Tibetan (2 %) (Pang et al., 2015[105]). South Asian populations such as Bangladeshi (4.7-11.2 % (Islam et al., 2013[59])), Sri Lankan (2.8-9.6 % (Topcu et al., 2002[137])) and Indian (1.5-8.9 % (Krishnakumar et al., 2012[67]; Nurfadhlina et al., 2006[98]) show a high frequency. Middle East populations such as Turkish (2.2 % (Takeshita et al., 2006[129])) and Iranian (0.9-2.5 % (Emamghoreishi et al., 2008[32]; Heravi et al., 2010[48])) show a lower frequency. However, the distribution is different in Caucasian populations: Nonspecific Caucasians had a frequency lower than 3 % (Audrain-McGovern et al., 2007[9]; Benowitz et al., 2006[13]; Fukami et al., 2006[39]; Malaiyandi et al., 2006[80][81]; Nakajima et al., 2006[91], 2004[95]; Rao et al., 2000[112]; Schoedel et al., 2004[117]; Xu et al., 2002[147]); Atlantic Europe populations such as Spanish (0.5-4 % (Oscarson et al., 1999[103]; Soriano et al., 2011[124])) and French (3.8 % (Gambier et al., 2005[42])) also have this allele; English populations such as British (0.3 % (Huang et al., 2005[54])), North American (1.6 % (Bloom et al., 2011[17])) and Canadian (0.2 % (O'Loughlin et al., 2004[99])) had a lower frequency; North of Europe populations such as Finish (1 % (Oscarson et al., 1999[103])) and Swedish (1.1 % (Djordjevic et al., 2013[31])) also report this allele. Southeastern Europe populations such as Serbian report a frequency of 2.9 % (Djordjevic et al., 2010[30]) and Tatar from Russia reported a frequency range from 6.8-16.9 % (Korytina et al., 2014[66]). African populations had a frequency less than 2 % and are reported as follows: Nonspecific African (≤1.9 % (Ho et al., 2009[49]; Nakajima et al., 2006[91]; Schoedel et al., 2004[117])), African American (0.5-0.6 % (Fukami et al., 2006[39]; Nakajima et al., 2004[95])), Ghanaian (2 % (Gyamfi et al., 2005[45])), Ethiopian (0.6 % (Aklillu et al., 2014[1])) and Namibian (0 % (Takeshita et al., 2006[129])). American populations show a variable frequency: Amerindian populations as Alaskan Yupik (14.5 % (Binnington et al., 2012[16])) have a higher frequency than Canadian natives (1 % (Schoedel et al., 2004[117])) and more than mestizo populations as Brazilian (0.5 % (Vasconcelos et al., 2005[139])), Ecuadorian (7.1 % (Soriano et al., 2011[124])), Chilean (3.7-4 % (Cáceres et al., 2012[20]; Roco et al., 2012[114])) and Hispanics (0 % (Benowitz et al., 2006[13])). On the other side of the world, the Māori native population from New Zealand report a high frequency (9.6 % (Lea et al., 2008[71])) of this allele and it is the only population reported in Oceania.

CYP2A6*5 is found in a higher frequency in Asian populations, East and Southeast Asian populations such as the Vietnamese had the highest frequency (14.6 % (Veiga et al., 2009[140])) compared with the Chinese (Liu et al., 2011[74]; Nurfadhlina et al., 2006[98]; Oscarson et al., 1999[103]; Schoedel et al., 2004[117]), Korean (Djordjevic et al., 2013[31]; Kwon et al., 2001[70]; Nakajima et al., 2006[91]; Yoshida et al., 2002[150]) and Malaysian (Nurfadhlina et al., 2006[98]) populations with a frequency less than 1.5 %. However, this allele is not found in the Japanese (Nakajima et al., 2001[92], 2006[91]; Schoedel et al., 2004[117]; Yoshida et al., 2002[150]); South Asians such as Indians show a frequency of less than 1 % (Krishnakumar et al., 2012[67]; Nurfadhlina et al., 2006[98]). Canadian populations such as the natives (0.5 % (Schoedel et al., 2004[117])) and Caucasians (0.1 % (Schoedel et al., 2004[117])) showed the minimum frequency. However, this allele was not found in the African, Caucasian, Middle East and American mestizo populations (Aklillu et al., 2014[1]; Djordjevic et al., 2013[31], 2010[30]; Gyamfi et al., 2005[45]; Huang et al., 2005[54]; Malaiyandi et al., 2006[80]; Nakajima et al., 2006[91]; Oscarson et al., 1999[103]; Rossini et al., 2006[115]).

CYP2A6*6 has been found only in a Japanese population study at a low frequency (0.4 % (Kitagawa et al., 2001[63])). It not has been found in African (Gyamfi et al., 2005[45]; Nakajima et al., 2006[91]), Caucasian (Malaiyandi et al., 2006[80]; Nakajima et al., 2006[91]), Asian (Nakajima et al., 2006[91]; Yoshida et al., 2002[150]) or Canadian native populations (Schoedel et al., 2004[117]).

CYP2A6*7 has a higher frequency in East and Southeast Asian populations such as Japanese (6.3-13 %) (Fujieda et al., 2004[36]; Fukami et al., 2005[38]; Minematsu et al., 2006[84]; Mwenifumbo et al., 2005[88]; Nakajima et al., 2006[91]; Schoedel et al., 2004[117]; Xu et al., 2002[147]; Yoshida et al., 2002[150]), Chinese (2.2-13.8 % (Liu et al., 2011[74]; Mwenifumbo et al., 2005[88]; Nurfadhlina et al., 2006[98]; Schoedel et al., 2004[117]; Xu et al., 2002[147]; Yuan et al., 2016[151])), Korean (3.6-11.1 % (Djordjevic et al., 2013[31]; Fukami et al., 2005[38]; Mwenifumbo et al., 2005[88]; Nakajima et al., 2006[91]; Yoshida et al., 2002[150])), Taiwanese (10 % (Mwenifumbo et al., 2005[88])), Thai (5-6.4 % (Mahavorasirikul et al., 2009[79]; Peamkrasatam et al., 2006[107])) and Malaysian (4.3 % (Nurfadhlina et al., 2006[98]; Yusof and Gan, 2009[152])) populations. It has also been reported in a lower frequency in Caucasian (≤0.3 % (Fukami, et al., 2005[38]; Malaiyandi et al., 2006[80]; Mwenifumbo et al., 2005[88]; Nakajima et al., 2006[91]; Schoedel et al., 2004[117]; Xu et al., 2002[147])) and Māori natives from New Zealand (1 % (Lea et al., 2008[71])). On the other hand, in Indian, African, Canadian native and Alaskan Yupik populations, this allele is not found (Binnington et al., 2012[16]; Fukami et al., 2005[38]; Gyamfi et al., 2005[45]; Mwenifumbo et al., 2005[88]; Nakajima et al., 2006[91]; Nurfadhlina et al., 2006[98]; Schoedel et al., 2004[117]).

CYP2A6*8 has a specific frequency in Asian populations such as Malaysian (4.2-5 % (Nurfadhlina et al., 2006[98]; Yusof and Gan, 2009[152]), Chinese (≤3.6 % (Nurfadhlina et al., 2006[98]; Schoedel et al., 2004[117]; Xu et al., 2002[147])), Japanese and Korean (≤2.2 % (Djordjevic et al., 2013[31]; Mwenifumbo et al., 2005[88]; Nakajima et al., 2006[91]; Schoedel et al., 2004[117]; Xu et al., 2002[147]; Yoshida et al., 2002[150])), Indian (0.9 % (Nurfadhlina et al., 2006[98])), Thai (≤0.5 % (Mahavorasirikul et al., 2009[79]; Peamkrasatam et al., 2006[107])) and is the lowest in Taiwanese (0.2 % (Mwenifumbo et al., 2005[88])). However, it has not been found in African, Caucasian, Canadian natives and Alaskan Yupik populations (Binnington et al., 2012[16]; Gyamfi et al., 2005[45]; Malaiyandi et al., 2006[80]; Mwenifumbo et al., 2005[88]; Nakajima et al., 2006[91]; Schoedel et al., 2004[117]; Xu et al., 2002[147]).

CYP2A6*9 is the most widely studied decreased function allele. Asian populations show the highest frequency: East Asian populations (Chinese, Korean and Japanese) had a frequency between 15-20 % (Benowitz et al., 2006[13]; Djordjevic et al., 2013[31]; Fujieda et al., 2004[36]; Liu et al., 2011[74]; Minematsu et al., 2006[84]; Nakajima et al., 2006[91]; Pitarque et al., 2001[110]; Schoedel et al., 2004[117]; Yoshida et al., 2003[149]; Yuan et al., 2016[151]), followed by South Asian population as Thai (12.1-20.4 % (Mahavorasirikul et al., 2009[79]; Peamkrasatam et al., 2006[107])) and Malaysian (10.4 % (Yusof and Gan, 2009[152])); Middle East populations as Turkish (6.9-7.2 % (Pitarque et al., 2001[110]; von Richter et al., 2004[142])), Iranian (12.4 % (Emamghoreishi et al., 2008[32])) and some ethnic groups among them (Sepehr et al., 2004[119]) such as Turkomans (14 %), Turks (5 %) and Zoroastrian Persian (4 %) showed a high frequency. Mediterranean European (Spanish (Soriano et al., 2011[124]) and Serbian (Djordjevic et al., 2010[30])), North European (Swedish (Djordjevic et al., 2013[31]; Pitarque et al., 2001[110])) and Central European (Hungarian (Fiatal et al., 2016[35])) populations show a frequency range of 5-8 %, which is the same as Caucasian (Audrain-McGovern et al., 2007[9]; Benowitz et al., 2006[13]; Haberl et al., 2005[46]; Malaiyandi et al., 2006[80][81]; Nakajima et al., 2006[91], 2004[95]; Schoedel et al., 2004[117]) and North American Caucasian populations (Bloom et al., 2011[17]; O'Loughlin et al., 2004[99]). A different pattern occurs in African populations; African and African American show a frequency of 7-10 % (Ho et al., 2009[49]; Mwenifumbo et al., 2008[87]; Nakajima et al., 2006[91], 2004[95]; Schoedel et al., 2004[117]), but in populations with a more conserved African component as Ghanaian (5.7 % (Gyamfi et al., 2005[45])) and Ethiopian (2.8 % (Aklillu et al., 2014[1])) the frequency is lower. Amerindian populations such as Canadian natives (15.5 % (Schoedel et al., 2004[117])) and Alaskan Yupik (8.9 % (Binnington et al., 2012[16])) had a heterogeneous frequency, which was the same as American mestizo populations such as Brazilian (5.7 % (Vasconcelos et al., 2005[139])), Ecuadorian (10.3 % (Soriano et al., 2011[124])), Mexican (16.4 % (Svyryd et al., 2015[126])) and other Hispanics (7.1 % (Benowitz et al., 2006[13])). The only population in Oceania to report this allele is the native population Māori from New Zealand with 19 % (Lea et al., 2008[71]).

CYP2A6*10 has been reported to be higher among Asian populations as Japanese (1-4.3 %) (Fujieda et al., 2004[36]; Mwenifumbo et al., 2005[88]; Nakajima et al., 2006[91]; Schoedel et al., 2004[117]; Xu et al., 2002[147]; Yoshida et al., 2002[150]), Chinese (0.4-4.3 % (Liu et al., 2011[74]; Mwenifumbo et al., 2005[88]; Nurfadhlina et al., 2006[98]; Schoedel et al., 2004[117]; Xu et al., 2002[147])), Korean (0.5-4.2 %) (Djordjevic et al., 2013[31]; Mwenifumbo et al., 2005[88]; Nakajima et al., 2006[91]; Yoshida et al., 2002[150]), Malaysian (4.3 %) (Nurfadhlina et al., 2006[98]), Taiwanese (4.1 %) (Mwenifumbo et al., 2005[88]) and Thai (1.6-2.4 %) (Mahavorasirikul et al., 2009[79]; Peamkrasatam et al., 2006[107]); and the Alaskan Yupik population with 1.9 % (Binnington et al., 2012[16]). However, Indian (Nurfadhlina et al., 2006[98]), Caucasian (Malaiyandi et al., 2006[80]; Mwenifumbo et al., 2005[88]; Nakajima et al., 2006[91]; Schoedel et al., 2004[117]; Xu et al., 2002[147]), African (Gyamfi et al., 2005[45]; Mwenifumbo et al., 2005[88]; Nakajima et al., 2006[91]; Schoedel et al., 2004[117]) and even Canadian native populations (Schoedel et al., 2004[117]) do not show any frequency.

CYP2A6*11 is not well-reported. However, it has been reported in a minimum frequency in Japanese and Korean populations at 0.5-0.7 % (Fujieda et al., 2004[36]; Nakajima et al., 2006[91]). The few analyzed African and Caucasian populations did not report this allele.

CYP2A6*12 has been reported at the highest frequency among the studied populations in Hispanics (3.5-4.7 % (Benowitz et al., 2006[13]; Koontz et al., 2009[65])) and Mexican (3.5 % (Borrego-Soto et al., 2015[18])) population. The frequency decreases in Caucasians (≤3 % (Audrain-McGovern et al., 2007[9]; Benowitz et al., 2006[13]; Haberl et al., 2005[46]; Koontz et al., 2009[65]; Malaiyandi et al., 2006[80][81]; Nakajima et al., 2006[91]; Schoedel et al., 2004[117])), Spanish (2.2 % (Oscarson et al., 2002[101])) and Canadian populations (1.1 % (O'Loughlin et al., 2004[99])). Then, in Asians, similar to in Middle East populations such as Iranians, it is reported in 1.3 % (Emamghoreishi et al., 2008[32]), but in East Asia only Japanese had a minimum frequency (0.8 % (Nakajima et al., 2006[91]; Schoedel et al., 2004[117])) while Chinese and Korean populations (Benowitz et al., 2006[13]; Koontz et al., 2009[65]; Nakajima et al., 2006[91]; Oscarson et al., 2002[101]; Schoedel et al., 2004[117]) did not report any frequency of the allele. Lastly, Amerindian populations such as Canadian natives and Alaskan Yupik reported a very low frequency (0.4-0.5 % (Binnington et al., 2012[16]; Schoedel et al., 2004[117])). In African populations, some studies show a low frequency (0.4 %) (Ho et al., 2009[49]; Schoedel et al., 2004[117]) and others do not show any frequency (Benowitz et al., 2006[13]; Koontz et al., 2009[65]; Mwenifumbo et al., 2008[87]; Nakajima et al., 2006[91]).

CYP2A6*13 has been reported in Japanese (1.1-1.5 % (Kiyotani et al., 2002[64]; Nakajima et al., 2006[91])) and Koreans (0.2 % (Nakajima et al., 2006[91])). It was not found in Caucasians and Africans (Kiyotani et al., 2002[64]; Nakajima et al., 2006[91]).

CYP2A6*14 has a higher frequency in Caucasian (3.5-5.2 % (Haberl et al., 2005[46]; Kiyotani et al., 2002[64]; Nakajima et al., 2006[91])) than in African (0.9-1.4 % (Mwenifumbo et al., 2008[87]; Nakajima et al., 2006[91])), but was not reported in Asians (Japanese and Korean) (Kiyotani et al., 2002[64]; Nakajima et al., 2006[91]).

CYP2A6*15 has been reported at a minimum frequency in Japanese (1.5-2.2 % (Kiyotani et al., 2002[64]; Nakajima et al., 2006[91])) and Korean (1.2 % (Nakajima et al., 2006[91])), but not in Caucasian and African populations (Kiyotani et al., 2002[64]; Mwenifumbo et al., 2008[87]; Nakajima et al., 2006[91]).

CYP2A6*16 has been reported in Caucasian (0.3-3.6 %) and African (0-1.7 %) populations (Kiyotani et al., 2002[64]; Mwenifumbo et al., 2008[87]; Nakajima et al., 2006[91]), but not in Japanese or Korean populations (Kiyotani et al., 2002[64]; Nakajima et al., 2006[91]).

CYP2A6*17 has a frequency of approximately 7.3-10.5 % in African populations (Fukami et al., 2004[41]; Ho et al., 2009[49]; Mwenifumbo et al., 2008[87]; Nakajima et al., 2006[91]), but not in Caucasian, Korean, Japanese or Alaskan Yupik (Binnington et al., 2012[16]; Fukami et al., 2004[41]; Nakajima et al., 2006[91]) populations.

CYP2A6*18 has a higher frequency range in Caucasian (0.3-2.2 % (Fukami et al., 2005[38]; Haberl et al., 2005[46]; Nakajima et al., 2006[91])) than in Korean (0.3-0.5 % (Fukami et al., 2005[38]; Nakajima et al., 2006[91])), but has not been found in Japanese and African populations (Fukami et al., 2005[38]; Nakajima et al., 2006[91]).

CYP2A6*19 has been reported in a low frequency in Korean (1-1.4 % (Djordjevic et al., 2013[31]; Fukami et al., 2005[38]; Nakajima et al., 2006[91])) and Japanese (0.5 % ), but not in Caucasian nor African (Fukami et al., 2005[38]; Nakajima et al., 2006[91]) populations.

CYP2A6*20 is present in African population at a frequency range between 1-1.7 % (Fukami et al., 2005[37]; Ho et al., 2009[49]; Mwenifumbo et al., 2008[87]; Nakajima et al., 2006[91]). This allele was not reported in Caucasian, Japanese and Korean populations (Fukami et al., 2005[37]; Nakajima et al., 2006[91]).

CYP2A6*21 has a higher frequency in Caucasian (0.5-2.3 %) (Al Koudsi et al., 2006[3]; Haberl et al., 2005[46]; Nakajima et al., 2006[91]) than in African populations (0.6-0.7 % (Mwenifumbo et al., 2008[87]; Nakajima et al., 2006[91])). It has not been reported in Japanese or Korean (Nakajima et al., 2006[91]) populations.

CYP2A6*22 has been reported at frequency less than 0.3 % (Haberl et al., 2005[2]; Nakajima et al., 2006[91]) in Caucasian populations, but not in Japanese or Korean (Nakajima et al., 2006[91]) populations.

CYP2A6*23 has a frequency range among 1-2 % (Ho et al., 2009[49], 2008[50]; Mwenifumbo et al., 2008[87]) in African populations, but was not reported in Caucasian, Japanese or Chinese populations (Ho et al., 2008[50]).

CYP2A6*24 has a frequency range among 0.7-1.3 % in African populations (Al Koudsi et al., 2010[2]; Ho et al., 2009[49]; Mwenifumbo et al., 2008[87]), but it has not been found in Caucasian, Chinese, Japanese and Taiwanese (Al Koudsi et al., 2010[2]) populations.

CYP2A6*25-*28 has only been reported in African populations, while CYP2A6*26-*27 has a frequency range of 0.7-0.9 % (Ho et al., 2009[49]; Mwenifumbo et al., 2008[87]) and CYP2A6*28 from 0.9-2.4 % (Ho et al., 2009[49]; Mwenifumbo et al., 2008[87]).

CYP2A6*31, *34 and *38 have not been studied in any population.

CYP2A6*35 has been reported at a frequency between 2.5-2.9 % (Al Koudsi et al., 2010[2]; Ho et al., 2009[49]) in African populations living in North American countries. In East Asian populations, such as Chinese, Japanese and Taiwanese, it has been found at a frequency of 0.5-0.8 % (Al Koudsi et al., 2010[2]), but it has not been found in Caucasian or Alaska Yupik (Al Koudsi et al., 2010[2]; Binnington et al., 2012[16]) populations.

CYP2A6*36 and *37 has been reported in Taiwanese populations at a frequency of 0.3 %, but not in African, Caucasian, Chinese nor Japanese (Al Koudsi et al., 2010[2]) populations.

CYP2A6*39 (0.6 %), *40 (0.2 %), *41 (1.2 %), *42 (0.2 %), *43 (0.2 %), *44 (0.2 %) and *45 (0.6 %) have only been reported in the African population (Piliguian et al., 2014[109]).

The gene duplication, CYP2A6*1X2, has been found at higher frequency in Asian populations as Asian (7.1 % (Benowitz et al., 2006[13])), Indian (3.5 % (Nurfadhlina et al., 2006[98])), Chinese (0.4-1.5 %) (Nurfadhlina et al., 2006[98]; Schoedel et al., 2004[117]; Xu et al., 2002[147]), Korean (≤0.2 %) (Fukami et al., 2007[40]; Nakajima et al., 2006[91]) and Malaysian (0.4 %) (Nurfadhlina et al., 2006[98]); the Hispanic population (3.5 %) (Benowitz et al., 2006[13]) has a higher frequency than the Ecuadorian population (0.5 %) (Soriano et al., 2011[124]); Caucasian populations have a frequency less than 2 % as in some Caucasian groups (≤1.7 %) (Benowitz et al., 2006[13]; Fukami et al., 2007[40]; Nakajima et al., 2006[91]; Rao et al., 2000[112]; Schoedel et al., 2004[117]; Xu et al., 2002[147]), Spanish (1.2 %) (Soriano et al., 2011[124]), Swedish (0.8 %) (Djordjevic et al., 2013[31]), Serbian (0.4 %) (Djordjevic et al., 2010[30]), North American (0.3 %) (Bloom et al., 2011[17]) and Canadian (0.2 %) (O'Loughlin et al., 2004[99]). In African populations, such African American (1.7 % (Fukami et al., 2007[40])) and Ethiopian (0.3 % (Aklillu et al., 2014[1])), this allele was present, but was not found in other African groups, Canadian natives or Alaskan Yupik (Binnington et al., 2012[16]; Nakajima et al., 2006[91]; Schoedel et al., 2004[117]).

Most studies address the majority of a country's population as a general population. However, a few studies focus on more specific population classifications as ethnic and regional groups. In the Asian population, it was studied among Tottori, Shimane, Ehime and Fukuoka people of the respective districts of Yonago, Izumo, Matsuyama and Kurume located in Japan (Takeshita et al., 2006[129]). In China, it has been compared in the prevailing Han Chinese group and the Uighur, Bouyei and Tibetan ethnic groups (Pang et al., 2015[105]). In the South of India, the frequencies of the people from Andhra Pradesh, Karnataka, Kerala and Tamil Nadu regions (Krishnakumar et al., 2012[67]) were compared. In Iran, some ethnic groups such as Turkomans from the Golestan Province, Turks from the Ardabil Province and Zoroastrian Persians from Tehran (Sepehr et al., 2004[119]) were studied. In Russia, the Tatar ethnic group was reported (Korytina et al., 2014[66]). In African populations, the ethnic group Ovambo from Namibia was reported (Takeshita et al., 2006[129]); and some ethnic groups such as Akan, Guan, Ewe, Ga, Nzima and Dargarti, but there were a few participants that were added in a single Ghanaian population (Gyamfi et al., 2005[45]). In Oceania, the Māori ethnic group from New Zealand is the only population reported across the continent (Lea et al., 2008[71]). In the American continent, the ethnic groups such as Yupik from Alaska and Canadian natives have been reported (Binnington et al., 2012[16]; Nowak et al., 1998[96]; Schoedel et al., 2004[117]).

## Conclusions and Perspectives

The enzyme responsible for metabolizing nicotine is mostly encoded by the *CYP2A6* gene, which is highly polymorphic. This variability is due to changes in DNA, which have generated different responses to nicotine and are reflected in the individual smoking behaviors along with other factors. It has been reported that this variability has been generated and distributed over a long time in different human populations, showing well defined ethnic patterns. Although association studies between carriers of certain variants and different smoking behaviors have been numerous with plausible results, population studies reporting frequencies of these variants are few. General population studies exhibit most reliable information than association studies with a variable frequency, because the population requirements are usually more specific, creating a population bias. However, it would be advisable to address this type of methodology in higher risk populations of smoking and those where policies to control smoking are less efficient, and where more smoking-related diseases are reported in the population. Additionally, the population must be characterized by more specific requirements, such as including the ancestry informative markers and avoiding "self-reporting" as the unique classification criteria. While there is a Human CYP-Allele Nomenclature Database in which the genetic findings of *CYP2A6* are unified, it is necessary to supplement it with updated data as per the population distribution. All of this could contribute to a personalized system that could detect, prevent and treat populations at risk of smoking, and in consequence, avoid tobacco consumption related diseases.
